# Membrane Contact Sites in Proteostasis and ER Stress Response

**DOI:** 10.1177/25152564251363050

**Published:** 2025-07-28

**Authors:** Febe Vermue, Aysegul Sapmaz, Ilana Berlin

**Affiliations:** 1 Oncode Institute and Department of Cell and Chemical Biology, 4501 Leiden University Medical Center, Leiden, Netherlands; 2 FLOW Protein Quality for Health, 4501 Leiden University Medical Center, Leiden, Netherlands

**Keywords:** proteostasis, endoplasmic reticulum, endolysosome, membrane contact sites, proteotoxic stress

## Abstract

Execution of all cellular functions depends on a healthy proteome, whose maintenance requires multimodal oversight. Roughly a third of human proteins reside in membranes and thus present unique topological challenges with respect to biogenesis and degradation. To meet these challenges, eukaryotes have evolved organellar pathways of protein folding and quality control. Most transmembrane proteins originate in the endoplasmic reticulum (ER), where they are subject to surveillance and, if necessary, removal through either ER-associated proteasomal degradation (cytosolic pathway) or selective autophagy (ER-phagy; organellar pathway). In the latter case, ER cargoes are shuttled to (endo)lysosomes – the same organelles that degrade cell surface molecules via endocytosis. Here, we provide an overview of dynamic coordination between the ER and endolysosomes, with a focus on their engagement in specialized physical interfaces termed membrane contact sites (MCSs). We cover how cross-compartmental integration through MCSs allows biosynthetic and proteolytic organelles to fine-tune each other's membrane composition, organization, and dynamics and facilitates recovery from proteotoxic stress. Along the way, we highlight recent developments and open questions at the crossroads between organelle biology and protein quality control and cast them against the backdrop of factor-specific diseases associated with perturbed membrane homeostasis.

## ER and Lysosomes – The Cradle and the Grave of Membrane Proteostasis

**Figure 1.**
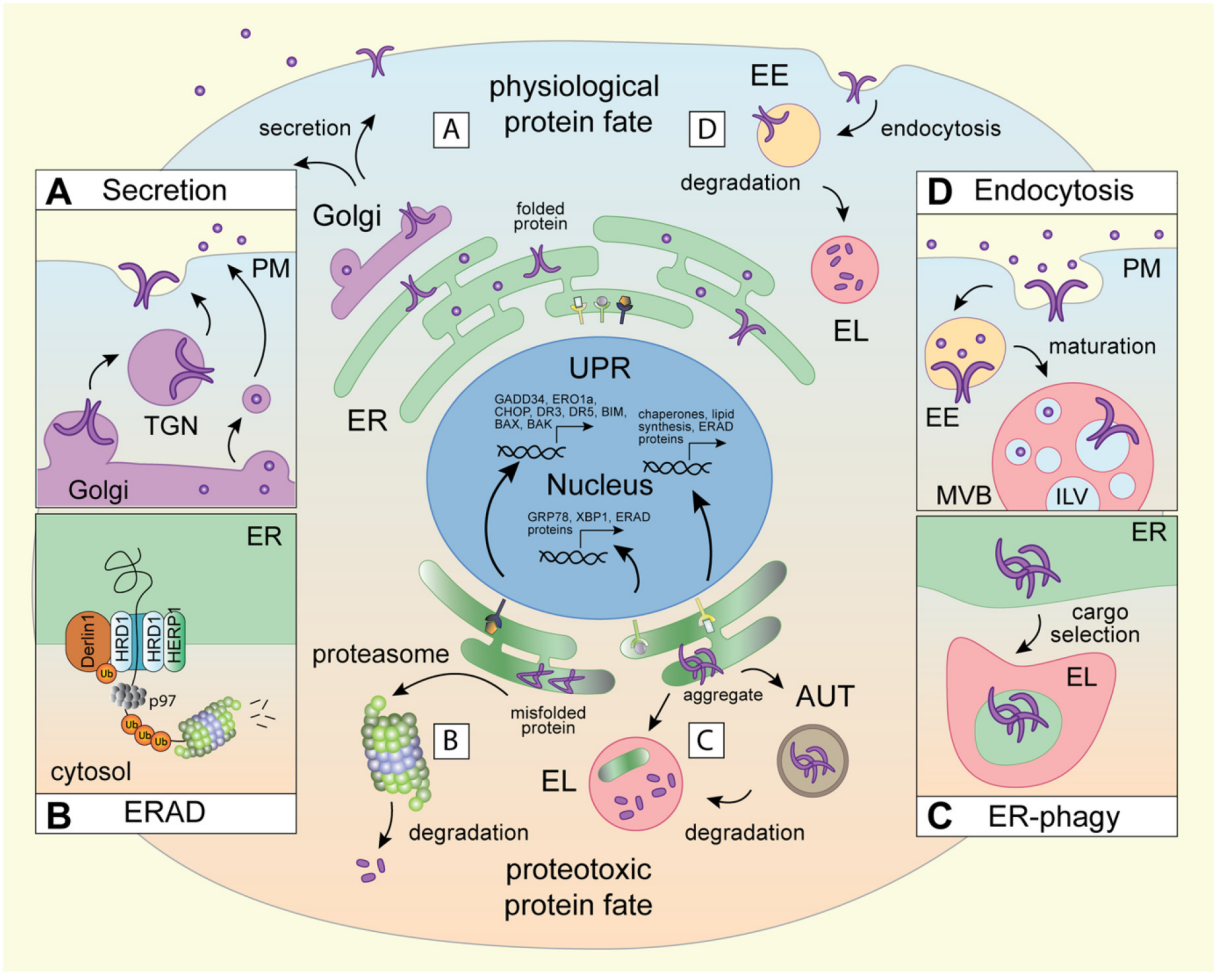
Biosynthetic and proteolytic membrane pathways in physiological and proteotoxic protein fate. The cell's membrane resident and extracellular proteins originate in biosynthetic organelles. A) The ER facilitates protein folding and assembly, while the Golgi apparatus oversees modifications and export of secretory and membrane-embedded cargoes to the cell surface and other membrane-bound compartments. B) Protein quality control at the ER is typically accomplished by the ERAD pathway. Here, the E3 ubiquitin ligase HRD1 and its cofactor Derlin-1, form a channel across the ER membrane and, in conjunction with the cytosolic ATPase p97, orchestrate retrotranslocation and polyubiquitination of misfolded proteins for proteasomal degradation (cytosolic route). Under conditions of ER stress, chaperones and ERAD machinery become upregulated downstream of the unfolded protein response (UPR) pathways (PERK, ATF6 and IRE1) to enhance cellular clearance capacity and restore homeostasis. C) Certain ER cargoes, including protein aggregates and damaged or extraneous ER membranes, are cleared by lysosomes via selective ER-phagy (organellar route), which operates either through intermediary autophagic compartments that fuse with lysosomes, or through direct feeding of ER membranes into the lysosomal lumen. D) Protein quality control at the plasma membrane operates via endocytosis, culminating in lysosomal degradation.

The term proteostasis refers to an equilibrium between protein synthesis, quality control and degradation. All proteins created by cells come into being on the ribosome, a macromolecular machine that translates mRNA transcripts of the genomic code into polypeptides (Wilson and Doudna Cate, 2012). Newly synthesized proteins intended for function in the cytosol or nucleosol become released into the aqueous milieu around them, where they receive folding assistance by local chaperones (Rosenzweig et al., 2019, Schopf et al., 2017). By contrast, proteins destined to reside at the cell surface, on organelles, or in extracellular space (i.e., extracellular matrix proteins (McCaughey and Stephens, 2019)) are co-translationally inserted into the membrane or lumen of the ER (Hegde and Keenan, 2022) (exceptions include mitochondrial residents utilizing dedicated insertion machinery on their home organelles (Pfanner et al., 2019)). Membrane and luminal proteins, which pass the ER surveillance check point, are allowed to proceed along the biosynthetic route to their intended site of function (Figure 1A), while those struggling to fold are reconsidered by the quality control machinery (McCaffrey and Braakman, 2016). Most integral membrane proteins produced in the ER end up at the cell surface, where they fulfill diverse roles in cell adhesion (Hynes, 1987), signal transduction (Deller and Yvonne Jones, 2000), intercellular communication (Jeppesen et al., 2023), host-pathogen interactions (Cossart and Helenius, 2014), and many others.

Threading of polypeptides through a lipid bilayer presents unique folding challenges, and membrane-embedded proteins, many of which possess multiple membrane-spanning segments, are notoriously prone to misfolding (Marinko et al., 2019, van der Sluijs et al., 2024). To prevent the accumulation of misfolded proteins, eukaryotes have evolved sophisticated clearance pathways (Santiago et al., 2020) converging either at the proteasome (cytosolic route) or the lysosome (organellar route). Proteasomes are proteolytic chambers present in the cytosol and the nucleoplasm designed to process one polypeptide chain at a time (Bard et al., 2018). (Endo)lysosomes, on the other hand, are acidic membrane-bound organelles that harbor active hydrolases for bulk digestion of proteins, lipids, nucleic acids and carbohydrates (Yang and Wang, 2021). Besides their digestive functions, lysosomes act as critical sensors of cellular metabolism and serve as platforms for transcriptional control (Savini et al., 2019). In mammalian cells, individual misfolded membrane and luminal cargoes are typically subject to proteasomal turnover via the ER-associated degradation pathway (ERAD) (Krshnan et al., 2022, Oikonomou and Hendershot, 2020) (Figure 1B). On the other hand, ERAD-resistant substrates, like antitrypsin-Z and mutant pro-collagen, as well as damaged or extraneous segments of the ER membrane, are diverted to ER-associated lysosomal degradation (ERLAD) (Salomo-Coll et al., 2025) via selective autophagy (Vargas et al., 2023) (Figure 1C). In other words, lysosomes directly impinge on proteostasis in the ER. Protein quality control from the plasma membrane also operates through the organellar clearance route – this time via endocytosis – where cell surface proteins become internalized into endosomes and escorted to lysosomes for degradation (Vietri et al., 2020) (Figure 1D). Biogenesis of endolysosomes, in turn, relies on trafficking from biosynthetic organelles (Saftig and Klumperman, 2009), and their behavior and function come under extensive influence of the ER membrane (Cohen et al., 2018). Collectively, the pathways governing both physiological and proteotoxic membrane protein fates reveal reciprocal interdependencies between the ER and the endolysosomal system, with broad implications for organelle homeostasis.

Considering the importance of balanced proteostasis to cellular and organismal health (Wilson et al., 2023, Lopez-Otin et al., 2023, Dufey et al., 2015), coordination of protein synthesis with degradation at the organellar level offers tangible benefits. In this review, we discuss the molecular underpinnings of this coordination and explore how it helps cells maintain organelle integrity, manage communication with their environment, and respond to proteotoxic stress. Our discussion centers around a rapidly evolving paradigm, wherein different intracellular compartments come together at specialized physical interfaces known as membrane contact sites (MCSs) to mediate material exchange and finetune each other's organization, dynamics, and function (Voeltz et al., 2024). In the following sections, we touch on the key architectural features of the ER and endolysosomes and discuss how these aspects are regulated through MCS formation. Alongside MCS, we also explore the role of ubiquitination – a versatile post-translational modification (PTM) with a small proteinaceous tag termed ubiquitin (Varshavsky, 2012) – as a common guiding principle for cargo selection across membrane protein quality control pathways, including ERAD (Christianson et al., 2023), endocytosis (Berlin et al., 2023), and various types of selective autophagy (Lamark and Johansen, 2021). Bringing these elements together, we consider how cross-compartmental interplay, in conjunction with ubiquitination and other PTMs, impacts organelle dynamics and membrane proteostasis, and ultimately disease pathogenesis.

### Architecture – Function Relationships

Organelle function is strongly linked to compartment architecture, localization, and dynamics. This connection is perhaps best illustrated by the multifunctional ER (Westrate et al., 2015), whose responsibilities extend beyond protein folding and quality control to include lipid production and storage (Jacquemyn et al., 2017), as well as calcium uptake and flux (Carreras-Sureda et al., 2018). To compartmentalize these activities, the greater ER network harbors spatially and morphologically distinct subdomains (Obara et al., 2023), where diverse membrane shaping molecules are used to create sheet-like (Sawyer et al., 2024) or tubular membrane profiles (Sandoz et al., 2023, Shen et al., 2019). Emerging from the nuclear envelope, the rough ER segment features densely packed membranes decorated with ribosomes, while the smooth ER consists predominantly of interconnected tubules that spread outwards to the cell periphery (Goyal and Blackstone, 2013). This perinuclear/peripheral dichotomy of the ER network is further accentuated in response to proteotoxic stress, which is defined as cellular damage resulting in the accumulation of misfolded proteins. To clear the damage and restore homeostasis, cells activate transcriptional programs of the three unfolded protein response (UPR) branches (IRE1, PERK and ATF6), resulting in the expansion of ER volume and upregulation of chaperones and degradation machinery (Hetz et al., 2020) (Figure 1). These elements are then spatially consolidated through concerted rearrangements of the perinuclear ER network into the ER quality control (ERQC) compartment (Wiseman et al., 2022). Formation of the ERQC compartment is orchestrated by the hairpin adaptor HERP1 (Leitman et al., 2014), whose expression is induced downstream of PERK (Kondratyev et al., 2007) (Figure 1B). Although spatially constrained, the ERQC remains dynamic (Benyair et al., 2015) and able to exchange cargoes with the peripheral ER through vesicular transport mechanisms (Ogen-Shtern et al., 2023). At the ERQC, heterodimers of the E3 ubiquitin ligase HRD1 and its cofactor Derlin-1 form a channel across the ER membrane (Wu et al., 2020). This complex, in collaboration with luminal chaperones (Behnke et al., 2015) and the cytosolic p97 ATPase (Braxton and Southworth, 2023), directs misfolded, or otherwise damaged, proteins for ubiquitin-dependent proteasomal degradation (Figure 1B). The intricacies of the ERAD pathway, both under physiological conditions and in the context of ER stress, have been extensively studied and excellent reviews on the topic abound, including a recent detailed overview of the mechanisms underpinning ERAD-mediated substrate processing (Christianson et al., 2023). We have therefore chosen to limit our discussion of ERAD to the context surrounding ER architecture and its interplay with proteolytic organelles.

Beyond ERAD, homeostasis of the ER membrane is broadly reliant on lysosomal turnover pathways (Gubas and Dikic, 2022). Many of these trafficking routes operate through intermediary autophagic compartments that sequester segments of the ER within their lumen and display membrane determinants necessary for fusion with lysosomes (macro-ER-phagy), while others entail direct feeding of ER membranes into the lysosomal lumen (micro-ER-phagy) (Reggiori and Molinari, 2022) (Figure 1C). Different flavors of ER-phagy become activated in response to starvation (An et al., 2019, Liang et al., 2020), aggregation within the ER lumen (Ji et al., 2019), ERAD dysfunction (Fasana et al., 2024), and upon recovery from ER stress (Loi et al., 2018). A diverse array of selective ER-phagy adaptors have been identified to date (e.g., FAM134B (Chen et al., 2022), ATL3 (Chen et al., 2019), RTN3 (Grumati et al., 2017), SEC62 (Fumagalli et al., 2016), and TEX264 (Chino et al., 2019), among others) that couple ER membranes to either autophagy modifiers (Khaminets et al., 2015) (LC3, GABARAP) or endocytic machineries (ESCRT) (Loi et al., 2019) for disposal in the lysosome. Beyond membrane shaping, certain ER-phagy receptors can directly bind misfolded luminal proteins, as shown for the ER stress induced protein CCPG1 (Ishii et al., 2023), thus providing an additional layer of selectivity in ER quality control. Together, these organellar clearance pathways help maintain ER morphology and homeostasis, while enabling the cell's largest endomembrane network to remain flexible in response to potential stressors.

Unlike the ER, the endolysosomal membrane system is discontinuous (i.e., vesicular). Yet, it still exhibits defined organization in cellular space (Neefjes et al., 2017). Endosomes arise when invaginations of the plasma membrane pinch off into the peripheral cytoplasm (Schiano Lomoriello et al., 2022) and fuse together to form early sorting compartments (Naslavsky and Caplan, 2018) (Figure 1D). As early endosomes mature through selective recycling (Cullen and Steinberg, 2018), gradual acidification and acquisition of late compartment identity (Scott et al., 2014), they travel along microtubule highways into the cell interior (Jongsma et al., 2023), where the bulk of proteolytic lysosomes resides (Johnson et al., 2016). The endolysosomal system employs phosphoinositides (Cullen and Carlton, 2012), and small GTPases (Borchers et al., 2021) that bind them, to distinguish between vesicles of different maturation states, which provides directionality to movement and cargo exchange. For instance, mammalian endolysosomes (and late autophagosomes) commonly carry GTPases Rab7, Rab2, and/or Arl8b that couple to specialized effectors for transport (Bonifacino and Neefjes, 2017) and cargo disposal through homotypic fusion (Lorincz et al., 2017, Ungermann and Moeller, 2025). The dynamic nature of the endosomal system, combined with the functional diversity of its members (Klumperman and Raposo, 2014), enables a high degree of responsiveness to environmental changes and fluctuating internal demands (Di Fiore and von Zastrow, 2014). As such, in the presence of extracellular inputs, the peripheral endolysosome contingent expands to accommodate the increasing need for signaling compartments (Villasenor et al., 2016). Conversely, under conditions of nutrient deprivation (Korolchuk and Rubinsztein, 2011) or proteotoxic stress (Bae et al., 2019), endolysosome move into the cell interior to boost degradation and facilitate return to homeostasis.

While organelle-intrinsic factors like membrane curvature and identity, together with active transport mechanisms (Bola and Allan, 2009, Bonifacino and Neefjes, 2017), are critical for maintaining the architectural landscape of the ER and the endolysosomal system individually, dynamic cross-compartmental interactions in the form of MCSs provide opportunities for coordination and cross-talk. The following sections discuss how MCSs between the ER and endolysosomes enable one organelle to steer the size, composition, and motility of the other, resulting in reciprocal modulation of membrane proteostasis.

### MCSs as Regulators of Organelle Architecture and Dynamics

**Figure 2.**
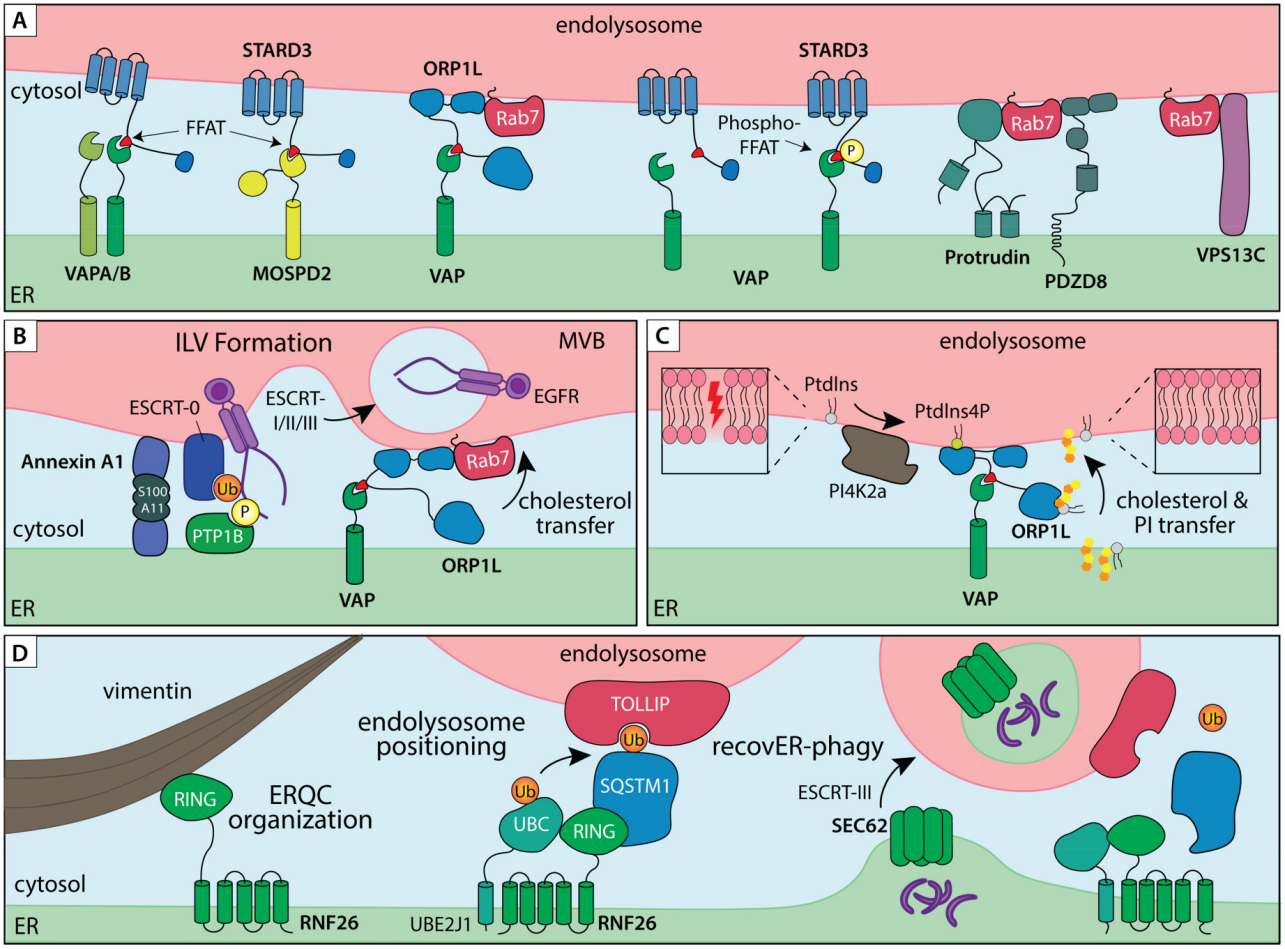
ER – endolysosome membrane contact sites in organelle dynamics and proteostasis. A) ER – endolysosome MCSs tethered by lipid transfer proteins and/or small GTPase effectors. ER tethers VAPA/B and MOSPD2 interact with FFAT peptide motifs of partner proteins residing on endosomal membranes, such as lipid transfer proteins STARD3 and ORP1L. Additionally, ER tethers Protrudin and PDZD8 interact with the small endosomal GTPase Rab7 to establish ER–endolysosome MCS involved in endosome transport. B) ER–endolysosome MCS controls multivesicular bodies (MVB) biogenesis and degradation of endocytic cargoes. Annexin A1, in complex with its cofactor S100A11, recruits the ER membrane embedded phosphatase PTP1B into an ER–endolysosome MCS, thereby coordinating dephosphorylation and ESCRT-mediated sorting of activated EGFR in intraluminal vesicles (ILVs) for degradation. The same contact site also couples VAP/ORP1L-dependent cholesterol transfer between the ER and proteolytic compartments. C) On-demand assembly of VAP/ORP1L MCS to mediated lipid transfer from the ER to endolysosomes during membrane repair. PI4K2a produces PI4P in response to damage in the endolysosome membrane, which recruits ORP1L for cholesterol and Phosphatidylinositol (PI) transfer. D) A ubiquitin-mediated ER–endolysosome MCS regulates endosome maturation and lysosomal ER turnover. A membrane embedded complex consisting of the E3 ligase RNF26 and E2 conjugating enzyme UBE2J1 is anchored in the perinuclear ER through direct interactions with Vimentin intermediate filaments. Ubiquitination of SQSTM1 in turn attracts endolysosomal membrane adaptors, such as TOLLIP, via their ubiquitin binding domains to position the endolysosomal repertoire in a perinuclear vesicle cloud. The same MCS complex facilitates disposal of extraneous or damaged ER fragments by lysosomes. Under conditions of ER stress, Vimentin and RNF26 help consolidate the ER quality control compartment (ERQC) in perinuclear space. During recovery from stress, shrinkage of the ERQC back to homeostatic levels via SEC62-dependent recovER-phagy pathway is facilitated by RNF26-mediated ER–endolysosome MCS.

Given its size and breadth of function, it is apt to consider the ER as the homeostatic hub of the eukaryotic cell. Living up to this title, the ER interacts and communicates with all other membranous compartments through various types of MCSs (Wenzel et al., 2022, Wu et al., 2018). Such physical interfaces commonly form when pairs of molecular tether proteins residing on different membranes bind one another, bringing their respective compartments into close apposition (<30 nm) (Scorrano et al., 2019) (though a growing number of singly tethered MCSs by bridge-like lipid transfer proteins has also been described (Reinisch et al., 2025)). The ER membrane is replete with MCS tethering proteins (Eisenberg-Bord et al., 2016), and ER MCSs involving members of the endolysosomal system, and its associated autophagic organelles, utilize tethers of diverse topologies and functions. Perhaps the best studied human integral ER membrane protein tethers, VAPA and VAPB (Murphy and Levine, 2016), are notorious for their ability to engage diverse MCS partners on other organelles (Murphy and Levine, 2016). These include lipid transfer proteins ORP1L (Rocha et al., 2009) and STARD3 (Alpy et al., 2013) on late endosomes and lysosomes (Figure 2A), and the retromer subunit SNX2 (Dong et al., 2016) on recycling compartments. A recent addition to the VAP MCS family, MOSPD2 (Di Mattia et al., 2018), also forms ER MCSs with endolysosomes, mitochondria and the Golgi, though its contribution(s) to the biology of these organelles is far less studied. MCS partnerships of VAP-family tethers are predicated on the recognition of conserved FFAT (two phenylalanines in an acidic tract) motifs by the Major Sperm Protein (MSP) domains (Neefjes and Cabukusta, 2021) (Figure 2A-C). Interactomes of different MSP domain-containing proteins exhibit aspects of redundancy (VAPA and VAPB share many binding partners) as well as selectivity (some MSPs prefer FFAT-like FFNT motifs (Cabukusta et al., 2023)), implying that flexible cross-compartmental interplay is advantageous to cellular homeostasis.

Advances in super-resolution time-lapse microscopy have revealed MCSs to be highly dynamic structures (Knedlik and Giacomello, 2024) that undergo rapid remodeling (Obara et al., 2024). Reversible PTMs, as well as local redox changes, can impart switchable controls to the formation and dissociation of MCS (Kors et al., 2022). This quality allows MCSs to function as temporally gated platforms, assembling transiently in response to acute demands, such as organelle damage, and dissolving once homeostasis is restored. One mode of on-demand tether pair engagement is enhanced binding affinity of MSP domains for phosphorylated FFAT motifs (Di Mattia et al., 2020) (by thus far unidentified kinases). Another example is rapid VAP-mediated MCS formation at sites of lysosomal damage, where local changes in lipid phosphorylation status produced by the phosphatidylinositol 4 kinase, PI4K2A (Radulovic et al., 2022) (Figure 2C). Here, direct delivery of cholesterol and phosphatidylserine from the ER restores the lysosomal membrane, in parallel to ESCRT-mediated repair. Ubiquitination can also promote reversible ER–endosome contacts, as evidenced by the ER-embedded ubiquitin ligase RNF26 (Figure 2D) and its opposing deubiquitinating enzyme USP15 (Jongsma et al., 2016). Spatial constraints provided to proteolytic organelles by RNF26 facilitate maturation of incoming endosomes and promote timely clearance of materials derived from extracellular space (Cremer et al., 2021, Jongsma et al., 2016). While detailed molecular networks operating at various ER MCSs remain to be elucidated, methodological developments for contact-dependent labeling (Cho et al., 2020) are setting the stage for future explorations into MCS composition, dynamics and function.

Within the greater endolysosomal system, propensity for MCS engagement is coupled to vesicle maturation state, where late endosomes and lysosomes appear far more likely to form and maintain contacts with the ER membrane than early endosomes (Friedman et al., 2013). Collectively, such MCSs serve as hubs for lipid exchange (Eden, 2016) and localized calcium signaling (Cremer et al., 2020), as well as provide the participating organelles with the means to influence each other's organization and motility. The ER moves throughout the cell by either sliding along microtubules or hitchhiking on other organelles, including endosomes and lysosomes (Guo et al., 2018). The latter mode of transport is afforded by MCSs and enables the ER network to reorganize along with changes in the movement of the endolysosomal system (Spits et al., 2021). Interactions with the ER can in turn alter the directionality of vesicle transport along microtubule tracks. Here, engagement of the tether, ORP1L, inhibits transport of late endosomes and mature autophagosomes into perinuclear space (Wijdeven et al., 2016), while binding of a hairpin tether Protrudin to Rab7 promotes processive motor loading for transport into the cell periphery (Raiborg et al., 2015) (Figure 2A). By affecting transport of mature endosomes, ER MCSs can support GTPase conversion from Rab7 to Arl8b, and ultimately Rab27a, to promote exosome secretion from MVBs (Verweij et al., 2022). In addition to controlling motility, ORP1L tethered MCSs serve as crucial platforms for cholesterol transfer between the ER and endolysosomes (Olkkonen and Ikonen, 2022). The same MCSs have recently been implicated in lysosomal membrane repair, which takes place in collaboration with the cholesterol-PtdIns4P transporter OSBP (Radulovic et al., 2022). ORP1L-mediated cholesterol transfer from the ER to MVBs also occurs at functionally distinct ER–MVB MCSs, tethered by Annexin A1 (Eden et al., 2016). Here, Annexin A1 recruitment, promoted through hetero-tetramerization with its Ca^2+^-dependent cofactor S100A11 (Rety et al., 2000), facilitates ILV formation in response to extracellular growth signals (Figure 2B). These examples highlight reciprocal influences exerted by biosynthetic and proteolytic organelles with respect to membrane composition and transport through dynamically assembled MCSs. In the following sections, we explore how this layer of spatiotemporal control converges with ubiquitination and ubiquitin-based recognition to guide the fate of membrane proteins.

## Ubiquitin and MCSs in Membrane Protein Quality Control

In striving to avert accumulation of unwanted proteins, eukaryotic cells have evolved robust mechanisms to label and retrieve proteins intended for degradation. Both proteasomal and lysosomal degradation pathways are dictated by ubiquitination (Pohl and Dikic, 2019). Covalent attachment of ubiquitin (typically to lysine (K) residues of target proteins) is mediated by a 3-step reaction cascade involving sequential engagement of an activating (E1), conjugating (E2) and ligating enzymes (E3) (Hershko and Ciechanover, 1998). A uniquely powerful aspect of ubiquitin (and ubiquitin-like) conjugation is that the above steps can be performed iteratively by exploiting previously attached ubiquitin moieties as new acceptor sites, resulting in a complex ubiquitin code (Yau and Rape, 2016). Furthermore, ubiquitination is reversible through the action of deubiquitinating enzymes or DUBs (Clague et al., 2019), endowing dynamic cellular events with temporal controls. Not surprisingly, proteostasis is strongly reliant on ubiquitin conjugation, both as a marker for protein degradation and as a means of regulating macromolecular complex assembly and function. Below we explore how these aspects come together to orchestrate quality control and degradation of the membrane proteome.

From the beginning of their life cycle, membrane-embedded proteins are subject to ubiquitin-dependent quality control. For most newly synthesized integral membrane proteins (like most luminal ones), this translates into ERAD-mediated surveillance, which ensures that misfolded proteins become ubiquitinated and targeted for proteasomal degradation. To accommodate high demand for ubiquitination, the ER membrane is replete with ubiquitin modifying machinery, including the ERAD conjugating enzymes UBE2J1 and UBE2J2 (Lenk et al., 2002), as well as E3 ligases HRD1 (Wu and Rapoport, 2018), RNF185 (El Khouri et al., 2013) and HERC3 (Kamada et al., 2024), among others. Overall, the ER is estimated to harbor a few dozen or more ubiquitin ligases (Fenech et al., 2020, Kaneko et al., 2016), including some whose cellular functions are presently unknown. Besides ERAD, ER-associated ubiquitination at scale could be useful in cargo selection for macro-ER-phagy, as recently demonstrated for the E3 ligase AMFR (Gonzalez et al., 2023). To achieve delivery of materials for lysosomal degradation via this pathway, cargoes of choice are incorporated into carrier vesicles capable of fusion with lysosomes (Perera and Zoncu, 2016). Generally, in autophagy, this is accomplished by coupling cargo ubiquitination to membrane remodeling events that drive the biogenesis of autophagosomes (Yin et al., 2020). In the context of ER-phagy, ubiquitination of ER-shaping proteins instead drives heterometic clustering and promotes cargo inclusion into autophagosomes (Foronda et al., 2023). In this capacity, AMFR ubiquitinates the ER-phagy receptor FAM134B within its reticulon homology domain, thereby promoting receptor clustering and stimulating ER-phagy (Gonzalez et al., 2023). Akin to the ER, the plasma membrane and the endolysosomal system embed (e.g., MARCH family ligases, RNF43, RNF167, ZNRF3 (Bauer et al., 2017, Bugter et al., 2024, Deshar et al., 2016)) or recruit (Cbl (Nikawa and Ishidoh, 2020) and ITCH (Aki et al., 2015), among others) ubiquitination machinery for protein quality control and degradation from these compartments. Although ubiquitination is not strictly required for endolysosomal degradation in mammals, where a number of ubiquitin-independent modes of cargo selection exist (MacDonald et al., 2015), it stimulates trafficking of extracellular cargoes to the lysosome (Piper et al., 2014). Along the way, the ER offers spatial and temporal guidance for key steps in endocytosis, with some examples highlighted below.

During endocytosis, invagination of the plasma membrane and maturation of vesicles through regulated membrane fusion and fission events dictate cargo flow. To accomplish these dynamic feats, endosomes enlist the support of local cytoskeletal networks for anchorage and provision of forces during membrane invagination (endosome biogenesis from the plasma membrane) and tubulation (endosome recycling) events. Such dynamic membrane processes are often coordinated by ER MCSs, as shown recently for tripartite contacts between the ER, plasma membrane and mitochondria during nascent endosome formation (Mesa et al., 2024). Recycling from endosomes is also subject to ER MCS control, where endosome-associated actin regulator Coronin 1C recruits the ER-embedded TMCC1 (Hoyer et al., 2018), thereby designating the location and timing of membrane fission (Rowland et al., 2014). Further along the endosomal maturation route, oversite from the ER becomes apparent once again in the biogenesis of multivesicular bodies – the key endolysosomal intermediates that serve as gateways to lysosomal degradation and exosome release (Arya et al., 2024, Hanson and Cashikar, 2012). MVBs form when ubiquitinated membrane cargoes are recognized and sequestered onto intraluminal vesicles (ILVs) of maturing endosomes by endosomal sorting complexes required for transport (ESCRTs) (Raiborg and Stenmark, 2009). Interestingly, the rate of cargo sorting into the lumen of MVBs can be influenced by the ER, as demonstrated for the epidermal growth factor receptor (EGFR) – a model cargo for endocytosis and lysosomal downregulation (Bakker et al., 2017). Upon arrival at the sorting endosome, activated EGFR is dephosphorylated by an ER-embedded protein tyrosine phosphatase 1B (PTP1B) in a step that strongly promotes receptor commitment for degradation (Eden et al., 2010). Recruitment of PTP1B to its EGFR substrate is facilitated by the leucine rich repeat kinase 1 (LRRK1) at ER–MVB MCSs tethered by Annexin A1 (Hanafusa et al., 2023), resulting in timely dephosphorylation and degradation of activated receptors. Annexin A1-positive MCSs also integrate ER-to-endosome cholesterol transport via the VAP–ORP1L axis (Eden et al., 2016) (Figure 2B), implying that lipid and protein homeostasis on proteolytic organelles are coregulated through on-demand formation (and dissolution) of ER MCS.

The functional diversity of ER MCSs with endolysosomes arises in part from the specialization of their molecular tethers, where ORP1L modulates vesicle transport and cholesterol flux, RNF26 scaffolds ubiquitin-dependent endolysosome positioning, and Annexin A1 coordinates lipid transfer with receptor sorting (among others), with each contributing distinct regulatory inputs into membrane homeostasis. In showcasing the broad influence of the ER over the endolysosomal system, the above scenarios illustrate how spatial concentration of multifunctional machineries at ER–endolysosome MCSs enables these interfaces to function not just as conduits, but as decision-making hubs for cargo triage. In keeping with the notion of reciprocity, below we consider how the same qualities are mobilized to drive organellar adaptations to proteotoxic stress in the ER and facilitate return to homeostasis.

## Cross-Compartmental Interplay in Organellar Adaptations to Proteotoxic Stress

As the primary site of folding and quality control for the membrane-embedded and secretory proteomes (Araki and Nagata, 2012), the ER serves on the front lines of sensing and responding to proteotoxic stress. Acute stress, which can be quantitatively assessed in experimental settings by monitoring protein aggregation through fluorescence signals emanating from Thioflavin T and 8-Anilino-naphthalene-1-sulfonic acid (Lee et al., 2020a), leads to a global expansion of the ER volume and segregation of misfolded proteins and damaged membranes with the ERAD machinery at the ERQC compartment. Here, ER chaperones calnexin and calreticulin recognize the unfolded protein regions and collaborate with the ERAD machinery to ensure their degradation (McCaffrey and Braakman, 2016). Efficient clearance of these proteotoxic species is crucial to preserving cell viability, as failures to resolve stress in a timely manner can trigger activation of apoptotic pathways (Wang and Kaufman, 2012). Formation of the ERQC is so fundamental to ER homeostasis that expansion of the ER through lipid biosynthesis can even alleviate ER stress independently of chaperone upregulation (Schuck et al., 2009). As clearance of the misfolded protein load draws to a close, restoration of ER homeostasis is further facilitated by scaling down of the ERQC. During this resolution phase of the ER stress response, lysosomal clearance mechanisms come critically into play, as discussed below.

Biogenesis and maturation of endosomes and lysosomes are central to ER membrane homeostasis, and disruptions in critical determinants of endosomal membrane remodeling, including ESCRT components and Rab7 function, have been shown to induce ER stress (Mateus et al., 2018, Oshima et al., 2016). Late endosomes and lysosomes respond to ER stress signals by moving into the ERQC compartment located in the pericentriolar space. This migration is stimulated in part by the ER transmembrane nuclease IRE1 (Calfon et al., 2002), whose activation induces degradation of the mRNA encoding the lysosome-related organelles subunit Blos1 (Bae et al., 2019). Establishment of the ERQC compartment, and retention of its associated proteolytic organelles are coupled through anchorage of both membrane systems on Vimentin intermediate filaments, predicated on direct interactions with proteins embedded in the ER membrane (Cremer et al., 2023) (Figure 2D). This organization helps cluster quality control machinery and provides spatial constraints for effective cross-compartmental interplay.

Given their high luminal acidity and presence of active proteases, it has long been thought that access to lysosomes is limited by homotypic membrane fusion, which allows only mature endocytic or autophagic compartments to deposit their cargoes inside degradative compartments (Luzio et al., 2014). However, during stress recovery, shrinkage of the perinuclear ER to homeostatic levels (a process known as recovER-phagy) is orchestrated by ESCRT-III-dependent piecemeal feeding of ER membranes into the lysosomal lumen (Loi et al., 2019). This process, coordinated by the ER receptor, SEC62 (Fumagalli et al., 2016), circumvents the formation of intermediary autophagic compartments and thus requires direct juxtaposition of ER membranes with proteolytic organelles. Here, the ER resident ubiquitination complex, comprised of UBE2J1 and RNF26, establishes ubiquitin-mediated MCS with endolysosomes (Cremer et al., 2021) to promote delivery of ERQC membranes for degradation (Cremer et al., 2023) (Figure 2D). UBE2J1, whose expression is upregulated under conditions of ER stress, is central to successful ER stress recovery, unlike its homologue UBE2J2 (Elangovan et al., 2017). Moreover, both UBE2J1 and RNF26 positively regulate ERQC formation as well as interact with HRD1 (Cremer et al., 2023, Lin et al., 2024), suggesting spatial coupling of proteasomal and lysosomal degradation pathways through ER-associated ubiquitin-dependent mechanisms. Furthering this notion, TOLLIP, one of the ubiquitin-binding cargo adaptors previously described to couple endolysosomes to RNF26 (Jongsma et al., 2016), has recently been found to target aberrant ER membrane proteins for lysosomal degradation, without requiring bulk ER turnover (Hayashi et al., 2023). Unlike canonical ER-phagy receptors, TOLLIP recognizes individual misfolded transmembrane polypeptides and delivers them to endolysosomes, providing a selective fail-safe mechanism, complementary to canonical ERAD. Additionally, RNF26 was recently identified as a protective factor against the buildup of nuclear condensates sequestering K48-linked polyubiquitinated proteins (Poch et al., 2025); however, the mechanism of action on the part of RNF26 in this context remains to be clarified. Taken together, these molecular insights intimate a complex and dynamic entanglement between diverse membrane-bound compartments under proteotoxic stress conditions and highlight interconnectivity between soluble and organellar protein quality control pathways in safeguarding cellular proteostasis.

## Disruptions in Proteotoxic Stress Responses are Associated with Disease

**Figure 3.**
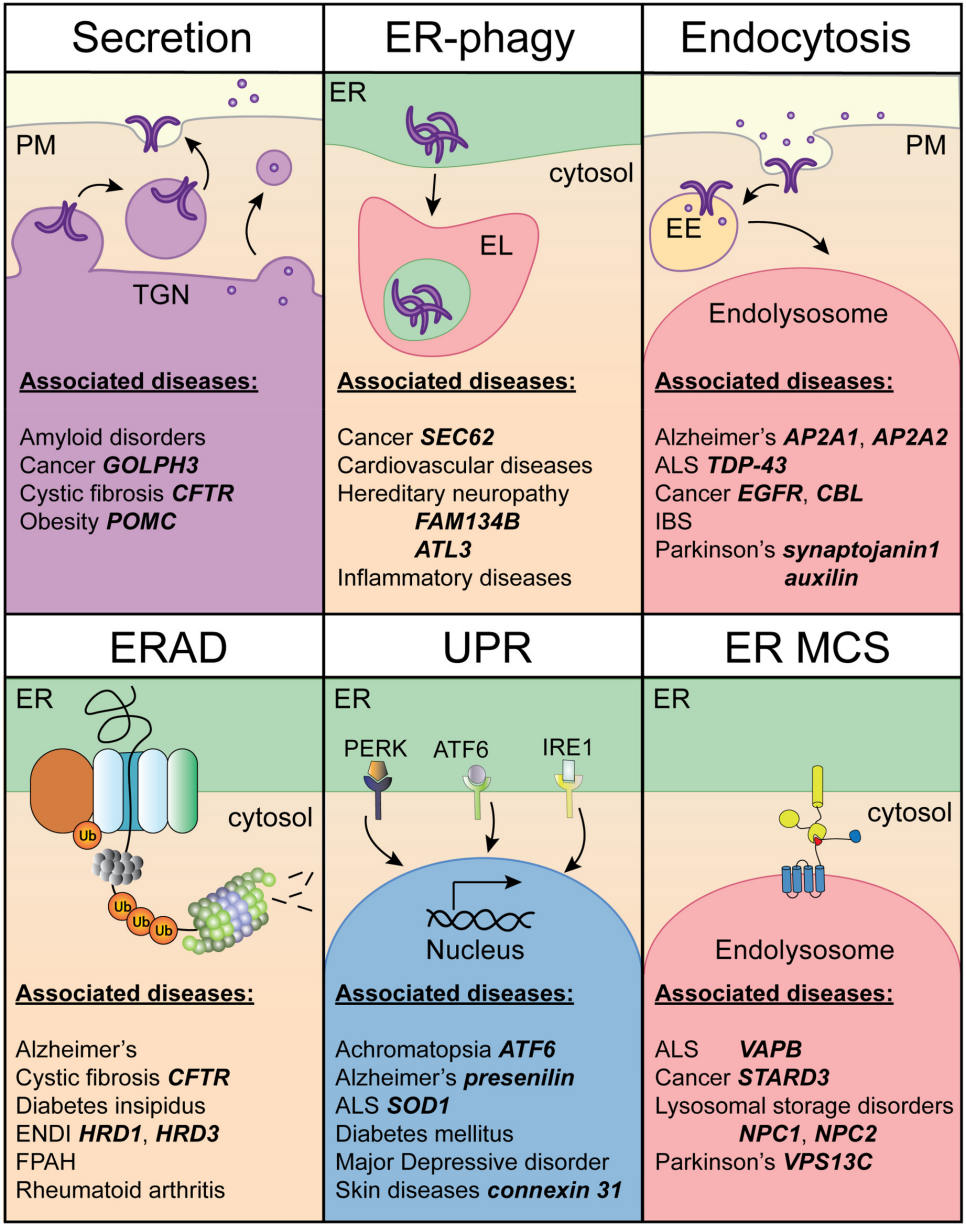
Overview of human diseases associated with disturbed membrane proteostasis. Examples of notable diseases per membrane-associated process category (Secretion, ERAD, ER-phagy, UPR, Endocytosis and ER MCS) are listed. Established mutations and/or alterations in expression levels of specific factors causative of the disease are indicated in bold.

Disruptions in cellular protein quality control pathways and responsiveness to proteotoxic stressors are intimately linked to a plethora of human ailments. Collectively, deregulation of ERAD (Singh et al., 2024), ER-phagy (He et al., 2021) and lysosomal functions (Udayar et al., 2022) accounts for a broad spectrum of neurological and neurodegenerative conditions, cancers and inflammatory and metabolic diseases (Figure 3), among others. While an exhaustive overview of these topics is beyond the scope of this review, it is useful to highlight examples of factor-specific diseases with clear links to faulty protein quality control in the context of membrane and organelle homeostasis. Among the best studied disease-causing secretion defects from biosynthetic organelles are mutations in the cystic fibrosis (CF) transmembrane conductance regulator (CFTR) (Ehre et al., 2023). In particular, the misfolded CFTR deletion mutant F508del is recognized and degraded by ERAD, resulting in loss-of-function phenotypes (Estabrooks and Brodsky, 2020). On the other hand, pro-opiomelanocortin (POMC) mutant C28F, implicated in early onset obesity (Creemers et al., 2008), evades ERAD-mediated degradation and becomes aggregated instead (Kim et al., 2018). Other conditions featuring altered ERAD processing include diabetes (Birk et al., 2009) and rheumatoid arthritis (Yagishita et al., 2012); ERAD-mediated clearance is also frequently co-opted in pathogen-host interactions (Guerriero and Brodsky, 2012). Alongside ERAD, dysregulation in selective removal of damaged or excess ER components by ER-phagy is broadly implicated in human pathologies spanning neurological and neurodegenerative disorders, various types of cancer, and metabolic diseases (Hubner and Dikic, 2020, Zhou et al., 2022). Numerous factor-specific defects map to various ER-phagy receptors and have been linked to disease development. For instance, mutations in FAM134B (Mo et al., 2020) and ATL3 (Krols et al., 2019) are associated with hereditary sensory neuropathy, while those found in RTN3 have been implicated in Alzheimer's disease (Zou et al., 2018). FAM134B G216R mutant protein, characterized by gain-of-function defects, has been reported to acquire enhanced propensity to oligomerize, resulting in excessive ER fragmentation and sensory neuron death (Jiang et al., 2020). On the other hand, in cancer, upregulation of selective autophagy pathways may confer an advantage, as evidenced by overexpression of SEC62 in various tumor types (Linxweiler et al., 2017).

A striking example of a factor-specific protein homeostasis disease, where diverse quality control and clearance pathways converge with membrane contact site biology is amyotrophic lateral sclerosis (ALS) (Webster et al., 2017) – a debilitating neurodegenerative condition affecting motor neurons (Mejzini et al., 2019). Amongst ALS-associated genes is the ER MCS tether VAPB (Borgese et al., 2021), whose P56S mutation adjacent to the FFAT binding site causes a rare dominantly inherited form of familial ALS. Mutant VAPB forms age-dependent ER-associated aggregates (Thulasidharan et al., 2024), which are thought to sequester wild type VAPB, thereby contributing to a loss of function in ER MCS formation. Moreover, VAPB aggregates may also be present in cells of patients with sporadic forms of ALS (Cadoni et al., 2020). While the resulting defects have largely been studied in the context of ER–mitochondria contacts and mitochondrial turnover, potential relevance of ER MCSs with endolysosomes to ALS pathogenesis remains to be explored. Here, VAPB aggregates may impair ER–endolysosome interactions, potentially disrupting stress-induced lipid homeostasis and clearance pathways, mechanisms increasingly recognized as central to neurodegeneration. Precedent for this exists with respect to lipid transfer between these organelles, as mediated by other tether proteins (van der Kant and Neefjes, 2014). As an example, loss of the bridge-like ER–lysosome tether, VPS13C, whose mutations are associated with early-onset Parkinson's disease (PD), causes aberrant lipid profiles on lysosomes (Hancock-Cerutti et al., 2022) and disrupts lysosomal damage repair (Wang et al., 2025). In a different context, mutations in Niemann-Pick type C (NPC) 1 and 2 genes give rise to disruptions in lipid transport at MCSs between lysosomes and other organelles, including the ER and mitochondria, leading to the accumulation of cholesterol and sphingolipids within lysosomes. Aberrantly composed and functioning MCSs have also been described in the cancer setting, where overexpression of STARD3 was found to overstabilize ER MCSs with MVBs, thereby inhibiting their progression to lysosomal proteolysis (Peretti et al., 2019). Collectively, the above examples emphasize the clinical relevance of cellular pathways underpinning membrane homeostasis and underscore the value of targeting organelle-associated dysfunction in therapy (Fernandez-Pereira et al., 2021).

## Conclusions and Perspectives

In this review, we have examined the nature of organellar proteostasis and its regulation through a complex and dynamic interplay between the ER and members of the endolysosomal system. Our discussion of this topic was focused on reversible physical contacts, formed through juxtaposition of membranes featuring distinct identities, as key mediators of reciprocal influence between different organelles. Collectively, the examples we chose to highlight above fall under a broader paradigm wherein maintenance of ER homeostasis relies on proteolytic organelles, and thus its intimate involvement in endocytic and autophagic flux comes as no surprise. As our understanding of how cross-compartmental interactions affect the flow of endocytic as well as ER-derived cargoes to proteolytic lysosomes through MCS continues to evolve, new questions come to the forefront. In the context of cellular responses to stress, these include whether ER MCSs affect the formation and/or function of stress-induced organelles, such as neuronal granulovacuolar degeneration bodies (GVBs), lipid droplets, or stress granules. A brief outlook along these lines is provided below.

GVBs are neuron specific vacuolar organelles induced by tau-associated pathology and characterized by a dense core containing endosomal and autophagic cargoes (Funk et al., 2011). Immunohistochemical analysis of post-mortem brains has demonstrated presence of UPR conduits inside the GVB lumen (Wiersma et al., 2019), and formation of these organelles is thought to promote cellular survival during proteotoxic stress. While the molecular mechanisms driving GVB formation remain unclear, their structural parallels to autolysosomes suggest that MCSs with the ER (and possibly other organelles) may be involved. Moreover, lipid droplets – organelles that emerge from the ER (Olzmann and Carvalho, 2019) – could contribute in this context, given their role in rebalancing lipid homeostasis (Velazquez et al., 2016). Along the same lines, dysfunction in lipid droplet turnover by endolysosomes (i.e., lipophagy) is implicated in ER stress (Garcia et al., 2021) and disease (Shin, 2020), and understanding their physical interplay with biosynthetic and proteolytic organelles in proteotoxic stress response may open new therapeutic possibilities.

The classical definition of MCS stipulates juxtaposition of two (or more) membranes, and thus implicitly excludes cross-compartmental interplay involving membraneless organelles. Interestingly, recent findings reveal engagement of ER MCSs with processing bodies and stress granules (Lee et al., 2020b), classified as phase-separated cytoplasmic compartments that sequester mRNAs together with their associated translation and degradation machineries (Luo et al., 2018). Because formation of these organelles is reversible, they can act as dynamic modulators of protein translation – a quality exploited by neurons to manage localized protein expression at synapses (Oh et al., 2013). Stress granules are specialized processing bodies that form in response to environmental stressors such as heat shock, which also induces protein misfolding. Moreover, formation of RNA-protein granules has been implicated in various degenerative disorders characterized by altered ribostasis, or post-transcriptional control (Ramaswami et al., 2013). Whether self-assembly of such structures is regulated through physical contacts with the ER membrane, and what relationship that may have to the proteotoxic stress response, is unknown.

To conclude our discussion on membrane protein homeostasis, it is fitting to step back and consider that globally, altered cellular proteostasis constitutes a hallmark of aging (Lopez-Otin et al., 2023), with clear links to disease. Therapeutic modulation of protein degradation using global (Manasanch and Orlowski, 2017) as well as targeted approaches (Paudel et al., 2023, Tsai et al., 2024) has therefore attracted considerable attention. In the context of cancer, high metabolic demands of cancer cells expose vulnerabilities to proteotoxic stress. This vulnerability is therapeutically exploited by proteasome inhibitors such as Bortezomib, used to treat multiple myeloma and mantle cell lymphoma (Sogbein et al., 2024). Conversely, in pathologies associated with protein aggregation, such as Alzheimer's and Parkinson's diseases, and certain forms of ALS, clearance pathways become overwhelmed, leading to the pursuit of therapeutic strategies that stimulate proteolytic capacity of neurons (Tabas and Ron, 2011). Current efforts to ameliorate proteotoxicity focus on enhancing quality control mechanisms by upregulating chaperones and augmenting proteosome activity, as well as controlling the capability of misfolded proteins to propagate to nearby cells. For protein aggregation diseases, where proteasomal clearance is technically challenging due to substrate insolubility, modulation of organellar degradation pathways, and their finetuning through cross-compartmental interplay, may prove fruitful. Still, key questions remain about how contact sites are spatially defined and temporally regulated, particularly in terms of recruitment versus exclusion of tethers, PTM machinery and signaling components to and from various types of MCSs. Understanding these principles will be instrumental for successfully targeting MCS-dependent pathways in cellular proteostasis and beyond.
